# CAV1 Exacerbates Renal Tubular Epithelial Cell Senescence by Suppressing CaMKK2/AMPK‐Mediated Autophagy

**DOI:** 10.1111/acel.14501

**Published:** 2025-01-30

**Authors:** Liya Sun, Lujun Xu, Tongyue Duan, Yiyun Xi, Zebin Deng, Shilu Luo, Chongbin Liu, Chen Yang, Huafeng Liu, Lin Sun

**Affiliations:** ^1^ Department of Nephrology, Key Laboratory of Kidney Disease and Blood Purification The Second Xiangya Hospital of Central South University Changsha Hunan China; ^2^ Department of Urology The Second Xiangya Hospital of Central South University Changsha Hunan China; ^3^ Guangdong Provincial Key Laboratory of Autophagy and Major Chronic non‐Communicable Diseases, Key Laboratory of Prevention and Management of Chronic Kidney Disease of Zhanjiang, Institute of Nephrology Affiliated Hospital of Guangdong Medical University Zhanjiang China

**Keywords:** AMPK, Autophagy, CaMKK2, CAV1, Kidney Aging, Renal Tubular Epithelial Cell

## Abstract

Renal proximal tubular epithelial cell (PTEC) senescence and defective autophagy contribute to kidney aging, but the mechanisms remain unclear. Caveolin‐1 (CAV1), a crucial component of cell membrane caveolae, regulates autophagy and is associated with cellular senescence. However, its specific role in kidney aging is poorly understood. In this study, we generated *Cav1* gene knockout mice and induced kidney aging using D‐galactose (D‐gal). The results showed that CAV1 expression increased in the renal cortex of the aging mice, which was accompanied by exacerbated renal interstitial fibrosis, elevated levels of senescence‐associated proteins γH2AX and p16^INK4a^, and increased β‐galactosidase activity. Moreover, autophagy and AMPK phosphorylation in PTECs were reduced. These phenotypes were partially reversed in D‐gal‐induced *Cav1* knockout mice. Similar results were observed in D‐gal‐induced human proximal tubular epithelial (HK‐2) cells, but these effects were blocked when AMPK activation was inhibited. Additionally, in CaMKK2 knockdown HK‐2 cells, si*CAV1* failed to promote AMPK phosphorylation, whereas this effect persisted when STK11 was knocked down. Besides, we examined the phosphorylation of CaMKK2 and found that si*CAV1* increased its activity. Given that CaMKK2 activity is affected by intracellular Ca^2+^, we examined Ca^2+^ levels in HK‐2 cells and found that D‐gal treatment reduced intracellular Ca^2+^ concentration, but *CAV1* knockdown did not alter these levels. Through GST pull‐down assays, we demonstrated a direct interaction between CAV1 and CaMKK2. In conclusion, these findings suggest that CAV1 exacerbates renal tubular epithelial cell senescence by directly interacting with CaMKK2, suppressing its activity and AMPK‐mediated autophagy via a Ca^2+^‐independent pathway.

## Graphical abstract

This study revealed that the expression of CAV1 is increased in the renal tissues of D‐gal‐induced aging mice and human renal proximal tubular epithelial cells (HK‐2). Using a GST pull‐down assay, it was demonstrated that CAV1 can directly bind to CaMKK2 and inhibit its activity, thereby suppressing the AMPK‐mediated autophagy process and exacerbating tubular epithelial cell senescence. This discovery provides new insights into the mechanisms of kidney aging.

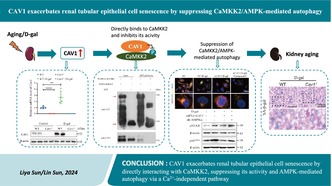

**Abbreviations**
AMPK: AMP‐activated protein kinase
CAMKK2: Calcium–calmodulin‐dependent protein kinase kinase 2
CAV1: Caveolin 1
Co‐IP: Co‐immunoprecipitation
IF: Immunofluorescence
MAP1LC3B/LC3B: Microtubule‐associated protein 1 light‐chain 3 beta
PRKAA: Protein kinase AMP‐activated catalytic subunit alpha
PTECs: Proximal renal tubule epithelial cells
SA‐β‐gal: Senescence‐associated β‐galactosidase
SQSTM1/p62: Sequestosome 1
STK11: Serine/threonine kinase 11
WT: Wild‐type
β‐actin: Actin beta

## 1 Introduction

Kidney aging is fundamentally driven by cellular senescence, which is closely linked to the progression of various chronic kidney diseases. Age‐related changes in the kidney include the loss of podocytes, glomerulosclerosis, as well as interstitial fibrosis, and tubular atrophy (Bolignano et al. 2014; Kubo et al. 2003). However, the underlying mechanisms remain largely elusive. Recent studies indicate that kidney aging is primarily characterized by alterations in the tubular interstitium rather than in the glomeruli (Martin and Sheaff 2007). Within the aging kidney, senescent cells are identified in both the medulla and cortex, with a predominant presence in the cortex, notably represented by proximal tubular epithelial cells (PTECs) (Valentijn et al. 2018). Senescent PTECs exhibit diminished repair capabilities and heightened secretion of pro‐inflammatory cytokines and extracellular matrix molecules, thereby exacerbating renal fibrosis, ultimately leading to a decline in renal filtration function (Luo et al. 2018). However, the precise mechanisms orchestrating these processes have yet to be fully elucidated.

Autophagy deficiency is a significant contributor to the senescence of renal PTECs. Studies have demonstrated that autophagy‐deficient PTECs in mice were found to have an accumulation of damaged mitochondria, ubiquitinated proteins, and SQSTM1/p62 (sequestosome 1)‐positive aggregates, which are associated with PTEC apoptosis and renal tubulointerstitial fibrosis (Kimura et al. 2011; Liu et al. 2012). The AMP‐activated protein kinase (AMPK) serves as a pivotal kinase modulating autophagy and exhibits ubiquitous expression across eukaryotic cells (Herzig and Shaw 2018; Liu et al. 2014). The complete activation of AMPK necessitates specific phosphorylation at the Thr172 site by upstream kinases such as calcium–calmodulin‐dependent protein kinase kinase 2 (CaMKK2), serine/threonine kinase 11 (STK11), and potentially other kinases (Ge et al. 2022; Hatsuda et al. 2023; Steinberg and Hardie 2023; Zhang et al. 2021). AMPK also plays a crucial role in kidney aging. Zhou Lili et al. reported significantly reduced levels of AMPK phosphorylation in PTECs from 24 months’ old mice compared to those from 2 months’ old mice (Zhu et al. 2022). Furthermore, the AMPK activator O304 was found to delay kidney aging by promoting energy metabolism and autophagy (Zhu et al. 2022). However, other molecules mediating AMPK activity in regulating autophagy and senescence remain to be identified.

Caveolin‐1 (CAV1) is a 22 kDa membrane protein and a key component of plasma membrane caveolae, playing a significant regulatory role in autophagy (Luo et al. 2021). Studies have indicated that autophagy is upregulated in CAV1‐deficient mouse endothelial cells, resulting in the attenuation of vascular inflammation and atherosclerosis (Zhang et al. 2020). Additionally, in colorectal cancer cells, CAV1 modulates autophagy by influencing AMPK activation (Ha and Chi 2012). Notably, CAV1 is predominantly expressed in glomeruli and renal tubular cells and is implicated in the initiation and progression of various renal disorders, including acute kidney injury (AKI), diabetic nephropathy, and IgA nephropathy (Emmerich et al. 2021; Luo et al. 2021; Moriyama et al. 2011). However, the role of CAV1 in kidney aging remains unexplored though some researchers have indicated that CAV1 is involved in the various cellular senescence processes, such as senescent human fibroblasts and bone marrow mesenchymal (Volonte and Galbiati 2020). It is still unclear whether CAV1 can regulate autophagy through AMPK to participate in renal aging.

In the present study, we observed elevated expression of CAV1 in the kidney of D‐galactose (D‐gal)‐induced mice, concomitant with reduced autophagy and AMPK protein phosphorylation in PTECs. Similar results were found in D‐gal‐treated HK‐2 cells. Subsequent in vitro studies demonstrated an interaction between CAV1 and CaMKK2, which inhibits AMPK activation via a Ca^2+^‐independent pathway, impairing autophagy and ultimately exacerbating the senescence of PTECs.

## 2 Results

### 2.1 Expression of CAV1 Is Significantly Upregulated in D‐gal‐Induced Aging Kidney

**FIGURE 1:**
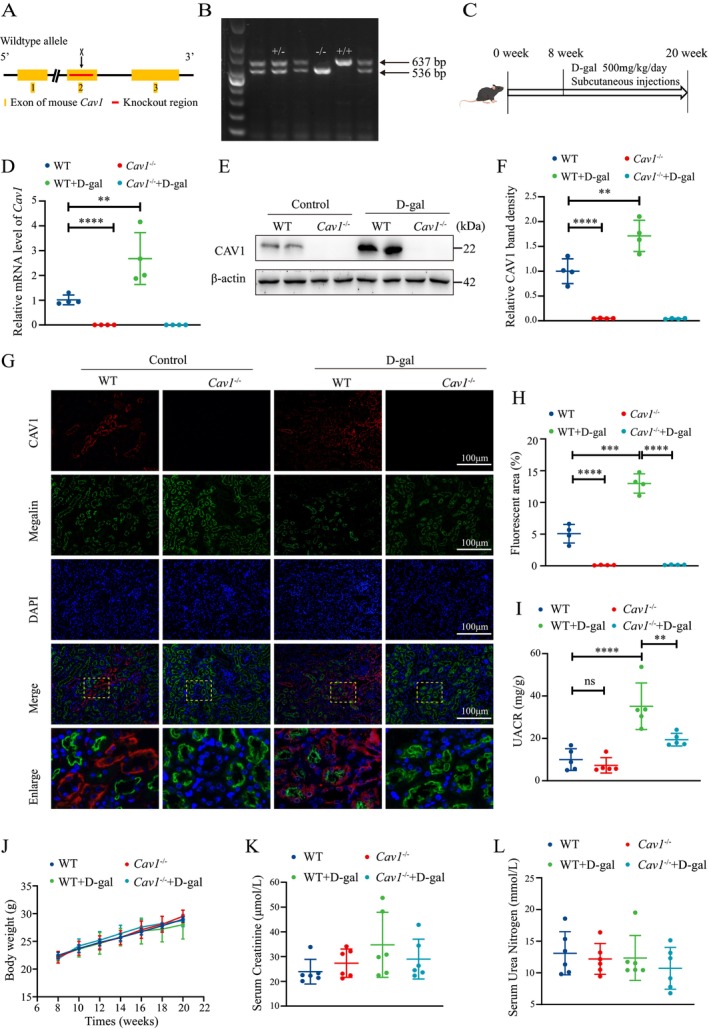
Upregulation of CAV1 expression in renal tissues of aging mice induced by D‐gal. (A) Schematic representation of the strategy for generation of Cav1 gene knockout mouse. (B) Genotyping of Cav1 by PCR amplification of mouse tail DNA and agarose gel electrophoresis. (C) Schematic diagram of the D‐gal‐induced aging mouse model, with continuous subcutaneous injections of D‐gal into the cervical dorsal region from 8 to 20 weeks of age. (D) qPCR analysis of the mRNA expression of *Cav1* in renal cortex tissues (*n* = 4). (E) Western blot analysis of CAV1 protein expression. (F) Semiquantitative analysis of CAV1 protein levels (*n* = 4). (G) IF staining analysis of CAV1 expression in the kidney: CAV1 (red), proximal tubule marker Megalin (green), and nuclei (blue). (H) Semiquantitative analysis of CAV1‐positive areas from the IF staining (*n* = 4). (I) UACR levels. (J) Changes in body weight of mice. (K) Serum creatinine levels. (L) Serum urea nitrogen levels. ****p < 0.0001, ***p < 0.001, **p < 0.01.

We utilized single‐cell RNA sequencing (scRNA‐seq) data to investigate changes in the expression of *Cav1* in PTECs of the mouse kidney during aging. These data were obtained from the Cell Landscape database (https://bis.zju.edu.cn/cellatlas/), spanning four time points from fetal, adult (8 weeks), 18 months, and 2 years. Our analysis revealed that *Cav1* expression was relatively low in the PTECs of adult mice, but it significantly increased in mice at 18 months and 2 years of age (Figure S1). To deeply investigate the role of *Cav1* in kidney aging, we constructed *Cav1* gene knockout mice. A schematic diagram of *Cav1* exon 2 knockout strategy is shown in Figure 1A. *Cav1* wild‐type (*Cav1* ^+/+^) and *Cav1* knockout (*Cav1* ^−/−^) mice were obtained by crossing *Cav1* heterozygous genotyped (*Cav1* ^+/−^) male and female mice. *Cav1* ^−/−^ mouse exhibited a single band at 536 bp, *Cav1* ^+/+^ mouse displayed a single band at 637 bp, while *Cav1* ^+/−^ mouse showed double bands at 536 bp and 637 bp (Figure 1B). Consistent with previous studies (Singh et al. 2018), male mice were subcutaneously injected with D‐gal at a dose of 500 mg/kg/day for 12 weeks starting at 8 weeks of age and weighing approximately 22 g to induce an aging mouse model (Figure 1C). Mice were divided into four groups: *Cav1* ^+/+^ (WT), *Cav1* ^−/−^, WT + D‐gal, and *Cav1* ^−/−^ + D‐gal. Quantitative PCR (qPCR) and Western blot confirmed the absence of expression of CAV1 in *Cav1* ^−/−^and *Cav1* ^−/−^ + D‐gal mice, while the mRNA and protein expression of *Cav1* were increased in the renal cortex of WT + D‐gal mice compared to the WT mice (Figure 1D–F). Tissue immunofluorescence (IF) co‐staining of CAV1 with the proximal tubule marker Megalin revealed a significant increase in the expression of CAV1 in the PTECs of WT + D‐gal mice compared to that of WT mice (Figure 1G,H). Furthermore, the urine albumin‐to‐creatinine ratio (UACR) in the WT + D‐gal group was significantly higher than that in the WT group, while the UACR was lower in the *Cav1* knockout group (Figure 1I). However, there were no significant differences in body weight, serum creatinine, and blood urea nitrogen levels observed among the groups (Figure 1J–L).

### 2.2 Deletion of the *Cav1* Gene Attenuates D‐gal‐Induced Renal Fibrosis and Aging‐Related Markers

**FIGURE 2:**
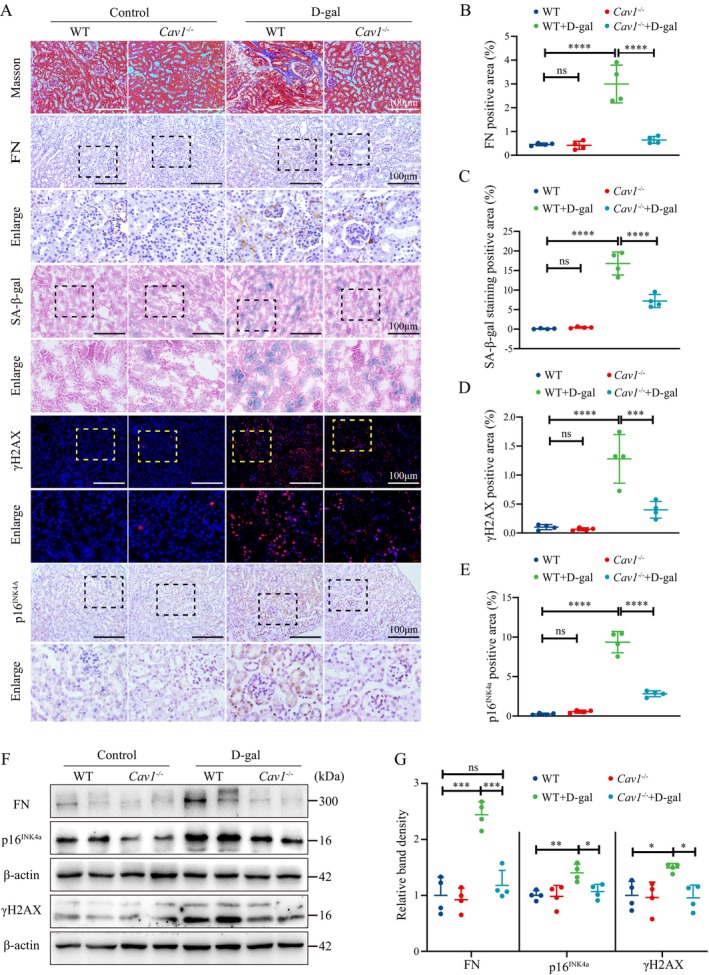
*Cav1* Deficiency alleviates D‐gal‐induced kidney aging. (A) Representative images of Masson and SA‐β‐Gal staining of renal tissues, IHC staining for FN and p16^INK4a^, and IF staining for γH2AX (Red fluorescence indicates γH2AX staining, blue fluorescence indicates nuclear staining). (B) Semiquantitative analysis of FN‐positive areas from the IHC staining in the kidney (*n* = 4); (C) Semiquantitative analysis of SA‐β‐Gal‐positive areas (*n* = 4). (D) Semiquantitative analysis of γH2AX‐positive areas from the IF staining (*n* = 4). (E) Semiquantitative analysis of p16^INK4a^‐positive areas from IHC staining (*n* = 4). (F) Representative western blot analysis of FN, γH2AX, and p16^INK4a^ protein expression in renal cortex. (G) Semiquantitative analysis of FN, γH2AX, and p16^INK4a^ protein expression relative to β‐Actin (*n* = 4); ****p < 0.0001, ***p < 0.001, **p < 0.01, *p < 0.05, ns indicates *p* > 0.05.

By Masson staining and tissue immunohistochemistry (IHC) for fibronectin (FN), increased notable deposition of interstitial collagen fibers and exacerbated interstitial fibrosis in the renal tissues of WT + D‐gal mice were seen compared to that of WT mice. Senescence‐associated β‐galactosidase (SA‐β‐gal) staining showed a significant increase in β‐galactosidase activity in the renal tissues of WT + D‐gal mice, along with the elevated expression of γH2AX and p16^INK4a^, as detected by IF and IHC. Conversely, *Cav1* ^−/−^ + D‐gal mice exhibited a reduction in these aging markers and phenotypes. No significant differences were observed between the WT and *Cav1* ^−/−^ groups (Figure 2A–E). In addition, compared to WT mice, an increased protein expression of FN, p16^INK4a^, and γH2AX in the kidney of WT + D‐gal mice was found, whereas it was decreased in the *Cav1* ^−/−^ + D‐gal group (Figure 2F,G). These results suggest that *Cav1* knockout mitigates renal aging‐related phenotypes.

### 2.3 *Cav1* Gene Deficiency Enhances Autophagy and Activates AMPK in PTECs of D‐gal‐Induced Mice

**FIGURE 3:**
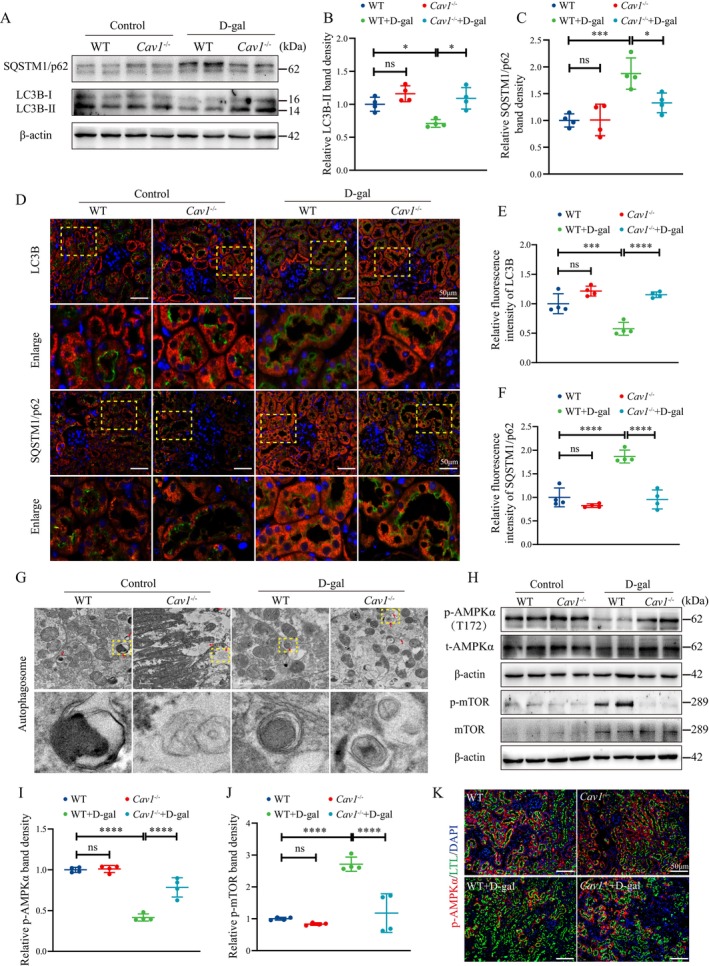
*Cav1* Knockout enhances autophagy and AMPK activity in PTECs of D‐gal‐induced aging mice. (A) Western blot analysis of LC3B‐II and SQSTM1/p62 protein expression in renal cortex tissues. (B) Semiquantitative analysis of LC3B‐II/β‐Actin protein expression (*n* = 4). (C) Semiquantitative analysis of SQSTM1/p62 protein expression (*n* = 4). (D) IF double staining of LC3B and SQSTM1/p62 with the proximal tubule marker Megalin, respectively: Red fluorescence indicates LC3B and SQSTM1/p62, green fluorescence indicates Megalin, and blue fluorescence indicates nuclei. (E) Semiquantitative analysis of the relative average fluorescence intensity of LC3B (*n* = 4). (F) Semiquantitative analysis of the relative average fluorescence intensity of SQSTM1/p62 (*n* = 4). (G) Transmission electron microscopy showing autophagosomes/autolysosomes in PTECs. (H) Western blot analysis of AMPK and mTOR and their corresponding phosphorylated proteins in the renal cortex. (I) Semiquantitative analysis of p‐AMPKα/β‐Actin protein expression (*n* = 4). (J) Semiquantitative analysis of p‐mTOR/β‐Actin protein expression (*n* = 4). (K) Representative IF double staining: Green for the proximal tubule marker LTL, red for p‐AMPKα fluorescence, and blue for nuclei. ****p < 0.0001, ***p < 0.001, *p < 0.05, ns indicates *p* > 0.05.

Western blot analysis showed that the autophagy‐related protein MAP1LC3B/LC3B‐ II (microtubule‐associated protein 1 light chain 3 beta II) was significantly decreased in the renal tissues of D‐gal‐induced mice, accompanied by an upregulation of SQSTM1/p62 expression. However, this alteration was reversed in *Cav1* gene knockout mice (Figure 3A–C). Furthermore, these results were confirmed by double staining of LC3B and SQSTM1/p62 with Megalin, respectively (Figure 3D–F). Transmission electron microscopy revealed that D‐gal substantially reduced the formation of autophagosomes in PTECs of WT mice, while this effect was reversed in the *Cav1* ^−/−^ + D‐gal mice (Figure 3G).

To further explore the relationship between impaired autophagy and renal tubular cell senescence, we treated D‐gal‐induced HK‐2 cells with chloroquine (CQ) to inhibit the autophagic flux. The results demonstrated that in D‐gal‐treated HK‐2 cells, the SQSTM1/p62 protein level was increased, while the LC3B‐II protein level was decreased, indicating the suppression of autophagy. Treatment with CQ alone also led to elevated expression of both p16^INK4a^ and γH2AX proteins. Moreover, when D‐gal and CQ were co‐interventions, the expression levels of p16^INK4a^ and γH2AX were further augmented compared to D‐gal treatment alone (Figure S2). These findings suggest that the inhibition of autophagy may exacerbate the senescence process of renal tubular epithelial cells.

Given the critical regulatory roles of AMPK and mTOR in cellular autophagy, we evaluated alterations in the activity of AMPK and mTOR. Compared to the WT group, significantly decreased p‐AMPKα and increased p‐mTOR levels in the WT + D‐gal mice were found, while these changes were partially reversed in the *Cav1* ^−/−^ + D‐gal group (Figure 3H–J). Double staining of p‐AMPKα and the proximal tubular marker *Lotus tetragonolobus* lectin (LTL) revealed a decrease in p‐AMPKα levels of PTECs in the WT + D‐gal mice, whereas an elevation was observed in the *Cav1* ^−/−^ + D‐gal group (Figure 3K).

### 2.4 *CAV1*‐siRNA Promotes Autophagy and Activates the AMPK–mTOR Pathway in D‐gal‐Treated HK‐2 Cells

HK‐2 cells were treated with D‐gal at concentrations of 100 mM and 200 mM for 72 h, followed by SA‐β‐gal staining and Western blot analysis. The results showed that with escalating concentrations of D‐gal treatment, HK‐2 cells displayed increasingly prominent morphological changes, characterized by enlarged and elongated features, alongside a heightened SA‐β‐gal positivity rate (Figure S3A). Furthermore, the expression of CAV1 protein exhibited a dose‐dependent increment (Figure S3B). Therefore, we selected 200 mM as the intervention concentration for subsequent experiments.

**FIGURE 4:**
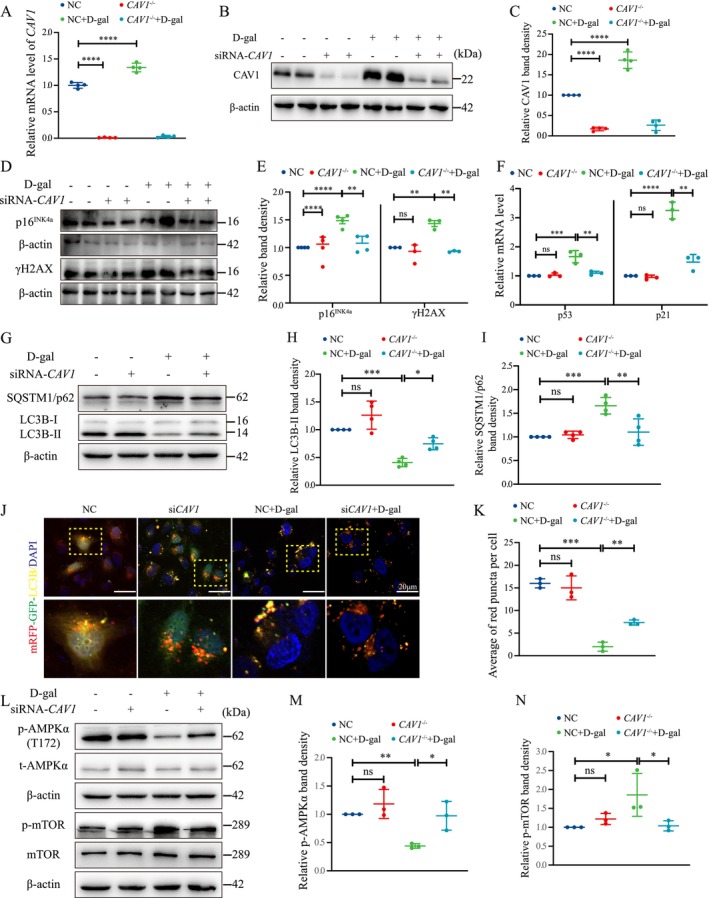
si*CAV1* Alleviates D‐gal‐induced HK‐2 cells senescence. (A) qPCR analysis of *CAV1* mRNA expression in D‐gal‐treated HK‐2 cells (*n* = 4). (B) Western blot analysis of CAV1 protein expression. (C) Semiquantitative analysis of CAV1 protein expression (*n* = 4). (D) Western blot analysis of p16^INK4a^ and γH2AX protein expression in D‐gal‐treated HK‐2 cells. (E) Semiquantitative analysis of p16^INK4a^ and γH2AX protein expression (*n* = 3 or 4). (F) qPCR analysis of the mRNA expression of p53 and p21 in D‐gal‐treated HK‐2 cells (*n* = 4). (G) Western blot analysis of LC3B and SQSTM1 protein expression in HK‐2 cells; (H) Semiquantitative analysis of LC3B‐II/β‐Actin in HK‐2 cells (*n* = 4). (I) Semiquantitative analysis of SQSTM1/p62 protein expression in HK‐2 cells (*n* = 4). (J) Autophagic flux analysis: HK‐2 cells were transfected with mRFP‐GFP‐LC3B plasmids for 24 h, followed by transfection with si*CAV1* or NC, and then treated with 200 mM D‐gal for 72 h: Autophagosomes appear as yellow dots, autolysosomes appear as red dots, and nuclei are stained with DAPI (blue). (K) Quantification of red fluorescent punctas per cell. (L) Western blot analysis of AMPK and mTOR phosphorylation levels. (M) Semiquantitative analysis of p‐AMPKα/β‐Actin protein expression (*n* = 3). (N) Semiquantitative analysis of p‐mTOR/β‐Actin protein expression (*n* = 3). ****p < 0.0001, ***p < 0.001, **p < 0.01, *p < 0.05, ns indicates *p* > 0.05.

HK‐2 cells were divided into four groups: Control (NC), *CAV1*‐siRNA (si*CAV1*), D‐gal (NC + D‐gal), and *CAV1*‐siRNA+D‐gal (si*CAV1* + D‐gal). Compared to the NC group, D‐gal significantly heightened *CAV1* mRNA and protein expression (Figure 4A–C). Similar results were corroborated by IF staining of CAV1 in HK‐2 cells (Figure S4). In addition, significantly upregulated protein levels of p16^INK4a^ and γH2AX, as well as the mRNA levels of p53 and p21, were observed in HK‐2 cells treated with D‐gal compared to the NC group (Figure 4D–F). Moreover, the NC + D‐gal group exhibited increased SA‐β‐gal‐positive cells (Figure S5). Nonetheless, all these alterations were substantially mitigated after si*CAV1* treatment.

Treatment of D‐gal to HK‐2 cells resulted in decreased LC3B‐II and increased SQSTM1/p62 protein expression (Figure 4G–I). Concurrently, a reduction in LC3B fluorescent puncta was observed, while the alteration was rescued by si*CAV1* (Figure S6). Furthermore, using the mRFP‐GFP‐LC3B double‐fluorescence system, it was observed that HK‐2 cells treated with D‐gal exhibited a reduction in both yellow and red puncta compared to the NC group, indicating decreased autophagic flux. However, this effect was partially reversed by si*CAV1* (Figure 4J,K). In addition, the altered expressions of AMPK and mTOR and their phosphorylation levels are consistent with in vivo experiments (Figure 4L–N).

### 2.5 Inhibition of AMPK Activation Blocked the Effects of *CAV1‐*siRNA on HK‐2 Cell Senescence and Autophagy

**FIGURE 5:**
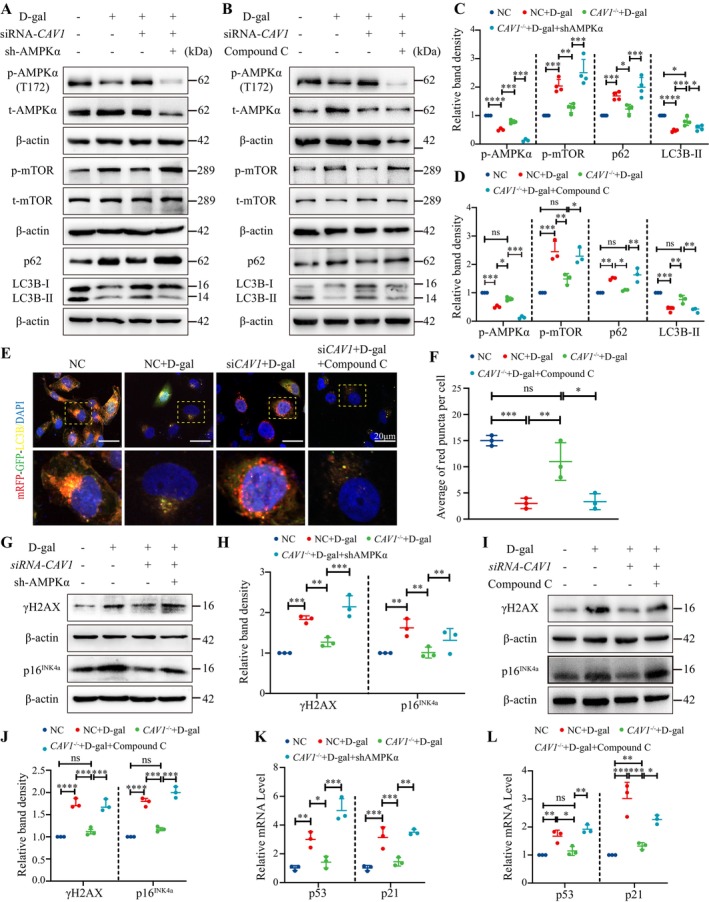
Inhibition of AMPK activation reverses the alleviating effects of si*CAV1* on D‐gal‐treated HK‐2 cells. (A) Western blot analysis of AMPK and mTOR phosphorylation, LC3B, and SQSTM1/p62 protein expression in AMPKα knockdown HK‐2 cells. (B) Western blot analysis of AMPK and mTOR phosphorylation, LC3B, and SQSTM1/p62 protein expression in HK‐2 cells treated with Compound C (4 μM) for 72 h. (C) Semiquantitative analysis of AMPK and mTOR phosphorylation, LC3B, and SQSTM1/p62 protein expression (*n* = 3) in (A). (D) Semiquantitative analysis of AMPK and mTOR phosphorylation, LC3B, and SQSTM1/p62 protein expression (*n* = 3) in (B). (E) Autophagic flux analysis using mRFP‐GFP‐LC3B fluorescence: Autophagosomes are indicated by yellow puncta, autolysosomes by red puncta, and nuclei stained with DAPI (blue). (F) Quantification of the average number of red dots per cell (*n* = 3). (G) Western blot analysis of γH2AX and p16^INK4a^ protein expression in AMPKα knockdown HK‐2 cells. (H) Semiquantitative analysis of γH2AX and protein p16^INK4a^ protein expression (*n* = 3) in (G). (I) Western blot analysis of γH2AX and p16^INK4a^ protein expression in Compound C treated‐HK‐2 cells. (J) Semiquantitative analysis of γH2AX and p16^INK4a^ protein expression (*n* = 3) in (I). (K) qPCR analysis of p53 and p21 mRNA expression in AMPKα knockdown HK‐2 cells (*n* = 3). (L) qPCR analysis of p53 and p21 mRNA expression in Compound C treated‐HK‐2 cells (*n* = 3). ****p < 0.0001, ***p < 0.001, **p < 0.01, *p < 0.05, ns indicated *p* > 0.05.

To further explore whether CAV1 contributes to cellular senescence by regulating the AMPK–mTOR pathway, we established an AMPKα‐knockdown HK‐2 cell line with stable transfection and treated the cells with Compound C, an inhibitor of AMPK phosphorylation. Our results demonstrated that both AMPKα‐knockdown and Compound C partially reversed the activation of the AMPK‐mTOR pathway by si*CAV1* in D‐gal‐treated HK‐2 cells, accompanied by the protein expression altered in LC3B and SQSTM1/p62 (Figure 5A–D). Additionally, si*CAV1* increased significantly the autophagic flux in D‐gal‐treated HK‐2 cells, but this effect was also partially inhibited by Compound C (Figure 5E,F). Furthermore, the downregulatory effects of si*CAV1* on the protein expression of γH2AX and p16^INK4a^, as well as the mRNA expression of p53 and p21, were partially reversed by AMPKα‐knockdown and Compound C (Figure 5G–L).

### 2.6 CAV1 Mediates AMPK Phosphorylation through Interaction with CaMKK2

**FIGURE 6:**
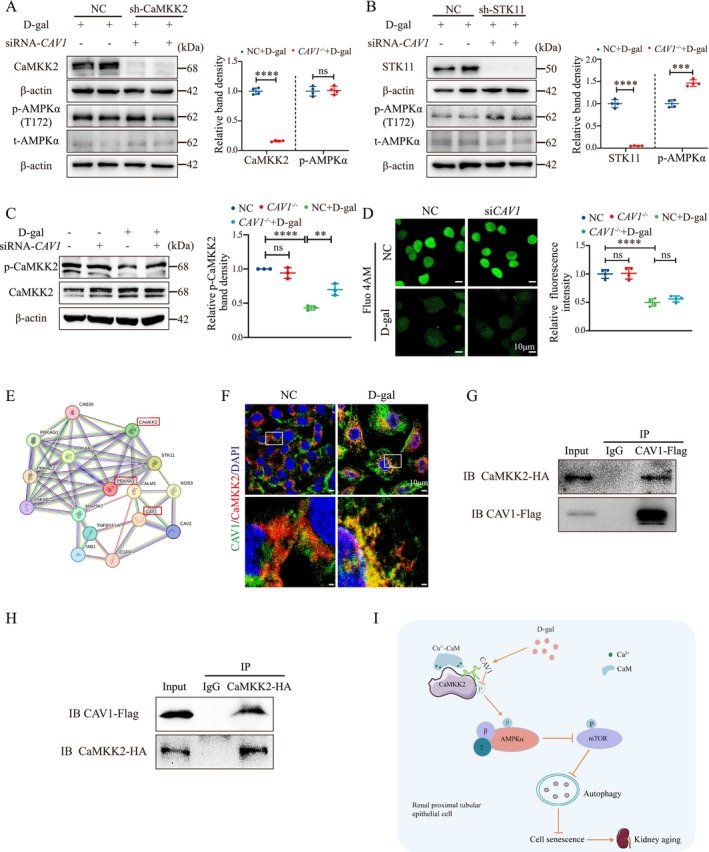
CAV1 directly interacts with CaMKK2 and inhibits the phosphorylation of CaMKK2 and AMPK via a Ca^2+^‐independent pathway. (A) sh‐CaMKK2‐stable HK‐2 cell line was constructed by transfecting HK‐2 cells with CaMKK2‐shRNA using the lipo3000 reagent with psPAX2 and pMD2.G as helper plasmids. The protein levels of CaMKK2, AMPK, and their phosphorylated forms were analyzed by Western blot. (B) Similarly, the sh‐STK11‐stable HK‐2 cell line was established, and the protein levels of STK11, AMPK, and their phosphorylated forms were assessed by Western blot. (C) Western blot detection of CAMKK2 phosphorylation. (D) Changes in intracellular Ca^2+^ concentration in HK‐2 cells were detected using the Fluo 4 AM Ca^2+^ fluorescent probe (4 μM), with Ca^2+^ indicated in green. (E) Prediction of potential interaction between CAV1 and CaMKK2 proteins using the STRING database. (F) Immunofluorescence co‐staining of CAV1 with CaMKK2 in HK‐2 cell. (G) Co‐IP was performed using a CAVl Flag antibody followed by Western blot analysis with a CaMKK2 HA antibody. (H) Co‐IP was performed using a CaMKK2 HA antibody followed by Western blot analysis with a CAVl Flag antibody. (I) CAV1 expression was upregulated in D‐gal‐induced senescent renal proximal tubular epithelial cells and directly interacted with CaMKK2 to inhibit AMPKα (Thr172) activity through a Ca^2+^‐independent pathway. This inhibition leads to increased phosphorylation of mTOR, reduced autophagy, and an accumulation of metabolic waste and damaged organelles, ultimately resulting in cellular senescence. Conversely, knockout of *CAV1* disrupts these processes, enhances autophagy, and thereby delays cellular senescence. ****p < 0.0001, ***p < 0.001, **p < 0.01, ns indicates *p* > 0.05.

CaMKK2 and STK11 serve as upstream kinases of AMPK, playing a pivotal role in the regulation of autophagy (Ge et al. 2022). To investigate whether CAV1 affects AMPK activity via CaMKK2 or STK11, we established stable HK‐2 cell lines with CaMKK2 or STK11 knockdown (sh‐CaMKK2 or sh‐STK11) and treated with D‐gal and si*CAV1*. Results revealed that the promotion of AMPK phosphorylation by si*CAV1* was found in the sh‐STK11 HK‐2 cell, while it was abolished in sh‐CaMKK2 HK‐2 cells (Figure 6A,B). Furthermore, we evaluated the phosphorylation levels of CaMKK2 and observed that in D‐gal‐treated HK‐2 cells, CaMKK2 phosphorylation was reduced, while it was increased following si*CAV1* treatment (Figure 6C). These findings suggest that si*CAV1* promotes AMPK phosphorylation in D‐gal‐treated HK‐2 cells through a CaMKK2‐dependent pathway.

Additionally, since CaMKK2 activation of AMPK is regulated by intracellular Ca^2+^ concentration (Marcelo, Means, and York 2016), we used the Fluo‐4 AM Ca^2+^ fluorescent probe to assess the changes in intracellular Ca^2+^ levels in HK‐2 cells. The results showed that D‐gal reduced Ca^2+^ concentration in HK‐2 cells, an effect not influenced by si*CAV1* (Figure 6D).

To further elucidate how CAV1 regulates the activity of CaMKK2, we first utilized the String database for protein–protein interaction prediction analysis. This analysis revealed a potential interaction between CAV1 and CaMKK2 (Figure 6E). To confirm this, we performed immunofluorescence co‐staining of CAV1 and CaMKK2 in HK‐2 cells, which showed clear co‐localization of the two proteins within the cell (Figure 6F). Next, we overexpressed CAV1‐Flag and CaMKK2‐HA plasmids in HK‐2 cells and performed co‐immunoprecipitation (Co‐IP) assays. The Co‐IP results strongly supported the interaction between CAV1 and CaMKK2 (Figure 6G,H). Finally, to further confirm the physical interaction between CAV1 and CaMKK2, we performed a GST pull‐down assay, which demonstrated that CAV1 directly binds to CaMKK2 (Figure S7). Together, these results suggest that CAV1 may modulate CaMKK2 activity through direct binding and interaction.

## 3 Discussion

In this study, we constructed a model of kidney aging in *Cav1* gene knockout mice induced by D‐gal and explored the role and mechanism of CAV1 in PTEC senescence for the first time. Our findings revealed that aging kidney exhibit heightened interstitial fibrosis and upregulated aging‐related markers, alongside diminished autophagy levels and AMPK phosphorylation in PTECs, while *Cav1* gene knockout partially ameliorated these phenotypes. Additionally, our results indicate that CAV1 directly binds to CaMKK2 and inhibits its activity through a Ca^2+^‐independent pathway, leading to reduced AMPK‐mediated autophagy and exacerbation of PTEC senescence (Figure 6I).

CAV1, a 22 kDa membrane protein, is intricately involved in the biogenesis of caveolae, maintaining their shape, structure, and functionality (Tang et al. 2021; Zhou et al. 2021). Moreover, CAV1 assumes a pivotal role in facilitating protein membrane targeting, endocytosis, signal transduction, and autophagy processes (Hou et al. 2021; Simón et al. 2020). Across various cell types, CAV1 also significantly contributes to cellular senescence (Volonte and Galbiati 2020). For instance, in corneal epithelial cells, CAV1 expression exhibits a progressive increase with age, with elderly individuals displaying nearly five times the number of caveolae compared to their younger counterparts (Rhim et al. 2010). Elevated CAV1 expression is similarly noted in the brains, spleens, and lungs of aged rats (Kang et al. 2006; Lim et al. 2010; Wicher, Prakash, and Pabelick 2019). Nevertheless, studies have demonstrated that CAV1 knockout promotes cellular senescence in quiescent human diploid fibroblasts and mouse embryonic fibroblasts (MEFs) (Volonte and Galbiati 2020). Consequently, CAV1 emerges as a pleiotropic regulator of cellular senescence, with its expression and functionality subject to variation across different organ‐specific and cellular pathophysiological contexts.

The role of CAV1 in kidney aging remains obscure, particularly its involvement in tubular cell senescence. In this study, we found that in WT mice, CAV1 predominantly localized in glomeruli, distal tubules, and vasculature, with minimal expression detected in PTECs, which is also consistent with the findings of previous research (Zhuang et al. 2011). However, in D‐gal‐induced mice, we observed an increase in CAV1 expression specifically in PTECs, along with a general upregulation in kidney tissues. Previous studies have also observed similar results in a bilateral renal ischemia–reperfusion mouse model, ureteropelvic junction obstruction, and atrophic proximal tubules within sclerotic kidneys (Krawczyk et al. 2017; Mahmoudi et al. 2003; Vallés et al. 2007). In summation, our results with others suggest a potentially significant role for CAV1 in PTEC injury and senescence.

The role of CAV1 in renal fibrosis remains incompletely understood, and existing studies present conflicting results. Research by Dhandapani et al. demonstrated that Caveolin‐1 Scaffolding Domain (CSD) peptides could reverse age‐related and angiotensin II (AngII)‐induced pathological changes in multiple organs, including renal fibrosis (Kuppuswamy et al. 2021). However, a study by Forrester et al. revealed that, in an AngII‐induced model, *Cav1* ^+/+^ mice exhibited more severe perivascular renal fibrosis and increased expression of vascular cell adhesion molecule‐1 (VCAM‐1) compared to *Cav1* ^−/−^ mice, which exacerbated vascular inflammation (Forrester et al. 2017). Furthermore, Mehta et al. (2021) found that CAV1 is essential for the synthesis of extracellular matrix proteins in glomerular mesangial cells (MCs). In *Cav1‐*deficient MCs, the expression of the antifibrotic protein follistatin is increased, suggesting that CAV1 knockout may have antifibrotic effects in renal cells. In this study, we observed that the deletion of the *Cav1* gene can ameliorate renal fibrosis. These contradictory results indicate the need for further in‐depth research to elucidate the specific mechanisms involved.

Previous research has firmly established the pivotal role of CAV1 as a critical regulator of autophagy. For instance, in CAV1‐deficient mouse endothelial cells, augmented autophagy mitigated vascular inflammation and atherosclerosis (Zhang et al. 2020). Moreover, CAV1 has been shown to competitively interact with the autophagy‐related protein 12 (ATG12)‐ATG5 system, thereby impeding its formation and function and consequently suppressing autophagy (Chen et al. 2014; Zhang et al. 2020). Autophagy deficiency significantly contributes to the senescence of PTECs. Cui et al. demonstrated impaired autophagy in the kidneys of aged rats, concomitant with the accumulation of polyubiquitin aggregates and damaged mitochondria (Cui et al. 2012). Yamamoto et al. reported that 24 months’ old mice with PTEC‐specific autophagy deficiency exhibited notable renal dysfunction and fibrosis, accompanied by mitochondrial dysfunction, mitochondrial DNA abnormalities, and nuclear DNA damage, the hallmark features of cellular senescence (Yamamoto et al. 2016). In our study, we also observed a marked reduction in autophagy in D‐gal‐induced senescent PTECs. However, this decline in autophagy was significantly reversed upon *CAV1* gene knockout. Our findings, in line with previous studies, underscore that the absence of CAV1 enhances autophagy in aging kidneys. Nevertheless, elucidating the precise mechanism warrants further exploration.

Previous investigations have delineated the dysregulation of nutrient‐sensing pathways in the PTECs of aged mice, characterized by diminished levels of the senescence‐associated protein SIRT1 and the inability to further activate AMPK despite mTOR overactivation (Yamamoto et al. 2016). Lili Zhou and collaborators found that the AMPK activator O304 ameliorated kidney aging by promoting energy metabolism and autophagy (Zhu et al. 2022). These findings underscore the involvement of the AMPK signaling pathway in kidney aging. In our study, we noted diminished AMPK phosphorylation in D‐gal‐induced PTECs, while *Cav1* knockout bolstered AMPK activation. By knocking down AMPKα and using Compound C to inhibit AMPK phosphorylation, we further corroborated that suppressing CAV1 expression enhances AMPK activation, augments autophagy, and retards PTECs senescence.

The mechanism by which CAV1 regulates AMPK phosphorylation remains unclear. In the present study, we observed that CAV1 influences AMPK phosphorylation through CaMKK2. CaMKK2 has been reported to phosphorylate downstream targets such as Ca^2+^/calmodulin‐dependent protein kinases I and IV (CaMKI and CaMKIV) and AMPK upon activation (Marcelo, Means, and York 2016). Under physiological conditions, the activation of CaMKK2 is regulated by changes in intracellular Ca^2+^ concentration, and it also exhibits significant autonomous activity (Marcelo, Means, and York 2016). The autonomous activity of CaMKK2 is restricted to CaMKI and CaMKIV, which are not regulated by Ca^2+^/calmodulin (CaM). However, for CaMKK2 to phosphorylate AMPK, the binding of Ca^2+^/CaM is still necessary (Tokumitsu and Sakagami 2022). This binding releases CaMKK2's autoinhibitory domain and induces a conformational rearrangement, enabling CaMKK2 to interact with AMPKα, forming a multiprotein complex comprising Ca^2+^/CaM, CaMKK2, and AMPKα (Green, Anderson, and Means 2011). If the activated conformation of CaMKK2 fails to form, it will be insensitive to Ca^2+^‐CaM stimulation (O'Brien et al. 2017). Here, we found a significant decrease in Ca^2+^ concentration in HK‐2 cells after D‐gal treatment. However, *CAV1* knockdown did not alter Ca^2+^ concentration. This information indicated that CAV1 influences the activity of CaMKK2 with a Ca^2+^‐independent pathway.

The CSD region of CAV1 interacts with various proteins such as Src family kinases, protein kinase A, and endothelial nitric oxide synthase (eNOS), among others, thereby regulating their activity and sequestering them within caveolae (Luo et al. 2021). We subsequently confirmed the direct interaction between CAV1 and CaMKK2 through GST pull‐down and Co‐IP assays. Based on these findings, we hypothesize that this interaction disrupts the conformational changes necessary for CaMKK2 activation, making it insensitive to Ca^2+^ stimulation and thereby impairing its activation. However, the precise mechanism remains to be further investigated.

In this study, we present the novel finding that CAV1 regulates CaMKK2 activity through direct binding, thereby inhibiting the AMPK‐mTOR signaling pathway and exacerbating renal tubular cell aging. This discovery unveils a previously unrecognized mechanism by which CAV1 modulates renal tubular cell senescence, offering new insights into the molecular mechanisms of kidney aging. In addition, our study also provides new targets for the diagnosis and treatment of age‐related kidney diseases.

## 4 Materials and Methods

### 4.1 Mice and Treatment

*Cav1* heterozygous mice (*Cav1* ^+/−^ mice, C57BL/6N background) were procured from Saiye Biotechnology. *Cav1* ^+/−^ male and female mice were cross‐bred to generate gene knockout mice (*Cav1* ^−/−^) and their littermate wild‐type mice (*Cav1* ^+/+^, WT) as controls. Genotypes were determined through PCR analysis of tail DNA using *Cav1*‐specific primer sequences: Forward primer 5′‐CTTGCAGCGCTGGAGTTTTCTG‐3′ and reverse primer 5′‐AACATGGAAAAGAGCTGTAACTGGAC‐3′. The mice were bred and maintained under specific pathogen‐free (SPF) conditions.

To establish an aging mouse model, 24 *Cav1* ^−/−^ mice and their littermate WT mice were divided into four groups: Wild‐type control group (WT group), Cav1 gene knockout group (*Cav1* ^−/−^ group), wild‐type modeling group (WT + D‐gal), and Cav1 gene knockout modeling group (*Cav1* ^−/−^ + D‐gal), consisting six mice in each group. For mice in the WT + D‐gal group and the *Cav1* ^−/−^ + D‐gal group, D‐gal was administered subcutaneously to the mice daily, commencing at 8 weeks of age, at a dosage of 500 mg/kg. Mice in the WT group and *Cav1* ^−/−^ group received equivalent volumes of normal saline injections. After 12 weeks of continuous injections, the mice were humanely euthanized, and their sera and kidneys were collected for subsequent experiments. Throughout the modeling period, the mice's body weight was monitored and recorded every 2 weeks. All animal experiments were approved by the Animal Ethics Committee at the Second Xiangya Hospital of Central South University.

### 4.2 Cell Culture and Treatment

HK‐2 was obtained from the American Type Culture Collection (ATCC) and cultured as previously described (Xiao et al. 2017). Transient transfection with siRNA was employed to knock down CAV1 in HK‐2 cells, while cellular senescence was induced by 200 mM D‐gal for 72 h. To elucidate the involvement of the AMPK pathway in D‐gal‐induced cellular senescence, HK‐2 cells were treated with 4 μM Compound C. Stable HK‐2 cell lines with the knockdown of CaMKK2, STK11, or AMPKα knockdown were generated via lentiviral transduction. Furthermore, following the manufacturer's instructions, Lipofectamine 3000 reagent (Invitrogen) was utilized for transfection of HK‐2 cells with *CAV1*‐siRNA, pcDNA3 CAV1(human) FLAG plasmid, and pCMV‐CaMKK2(human)‐3 × HA‐Neo plasmid. The siRNA for *CAV1* is 5′‐CCACCTTCACTGTGACGAA‐3′, shRNA for CaMKK2 knockdown is 5′‐GTGAAGACCATGATACGTAAA‐3′, shRNA for STK11 is 5′‐GCCAACGTGAAGAAGGAAATT‐3′, and shRNA for AMPKα is 5′‐GAAGGTTGTAAACCCATATTA‐3′.

### 4.3 Reagents and Antibodies

The primary reagents used in this study were obtained as follows: D‐galactose (G0750) from Sigma; DMEM, F12, and fetal bovine serum from Gibco; Compound C (HY‐13418) from MCE; and the proximal tubule marker *Lotus tetragonolobus* lectin (LTL) from Vector Laboratories (FL‐1321‐2). The primary antibodies used in the experiments included anti‐CAV1 (Cell Signaling Technology, 3238s), anti‐p14^INK4a^ (Santa Cruz Biotechnology, sc‐1661), γH2AX (Abcam, ab26350), anti‐LC3B (Cell Signaling Technology, 3868s), anti‐LC3B (Proteintech, 18725‐1‐AP), anti‐SQSTM1/p62 (Servicebio, GB11531‐100), anti‐p‐PRKAA/AMPKα (Cell Signaling Technology, 2535), anti‐t‐PRKAA/AMPKα (Gene Tex, GTX50705), anti‐p‐mTOR (Cell Signaling Technology, 5536T), anti‐t‐mTOR (Cell Signaling Technology, 2972s), anti‐STK11/LKB1 (Santa Cruz Biotechnology, sc‐32245), anti‐CaMKK2 (Proteintech, 11549‐1‐AP), anti‐Flag (ThermoFisher Scientific, MA1‐91878), and anti‐HA (Proteintech, 81290‐1‐RR). The secondary antibodies used were HRP‐conjugated Affinipure Goat Anti‐Mouse IgG (Proteintech, SA‐00001‐1), HRP‐conjugated Affinipure Goat Anti‐Rabbit IgG (Proteintech, SA‐00001‐2), goat anti‐rabbit secondary antibody for immunohistochemistry (Servicebio, G1213), goat anti‐mouse secondary antibody for immunohistochemistry (Servicebio, G1214), Goat Anti‐Rabbit IgG H&L (Alexa Fluor 488) (Abcam, ab150077), and Goat Anti‐Mouse IgG H&L (Alexa Fluor 594) (Abcam, ab150116).

### 4.4 Western Blot Analysis

Total protein was extracted from the renal cortex or HK‐2 cells. The protein concentration was determined using a BCA protein assay kit (TaKaRa, T9300A). Subsequently, total protein samples were separated by SDS‐PAGE and transferred onto a PVDF membrane (Millipore). After blocking, the membrane was incubated overnight at 4°C with primary antibodies, followed by incubation at room temperature for 1 h with the appropriate secondary antibodies. Protein expression levels were visualized using Super ECL Plus (S6009L, US Everbright Inc) and quantified using ImageJ software (National Institutes of Health, USA).

### 4.5 Real‐Time Quantitative PCR

Total RNA was extracted from renal cortex tissue and HK‐2 cells using TRIzol reagent. The RNA was then reverse‐transcribed into cDNA using the Accurate Biology Reverse Transcription Kit (Accurate Biology, AG11728). Subsequently, RT‐qPCR was conducted with the SYBR Green Premix Pro Taq HS qPCR Kit (Accurate Biology, AG11701) on the 7300 Real‐Time PCR System (Applied Biosystems). The primer sequences used for RT‐qPCR are as follows: For mouse *Cav1* (forward primer 5′‐GGAACAGGGCAACATCTACAA‐3′ and reverse primer 5′‐GACCAGGTCAATCTCCT‐3′), for mouse *ACTB* (forward primer 5′‐GGCTGTATTCCCCTCCATCG‐3′ and reverse primer 5′‐CCAGTTGGTAACAATGCCATGT‐3′), for human *CAV1* (forward primer 5′‐GCGACCCTAAACACCTCAAC‐3′ and reverse primer 5′‐ATGCCGTCAAAACTGTGTGTC‐3′), for human *p53* (forward primer 5′‐CAGCACATGACGGAGGTTGT‐3′ and reverse primer 5′‐TCATCCAAATACTCCACACGC‐3′), for human *p21* (forward primer 5′‐TGTCCGTCAGAACCCATGC‐3′ and reverse primer 5′‐AAAGTCGAAGTTCCATCGCTC‐3′), and for human ACTB (forward primer 5′‐CCTGGCACCCAGCACAAT‐3′ and reverse primer 5′‐GGGCCGGACTCGTCATAC‐3′).

### 4.6 Immunohistochemical Staining

Paraffin‐embedded renal tissue sections, approximately 4 mm thick, underwent a series of processing steps including deparaffinization, hydration, antigen retrieval, and peroxidase blocking. Subsequently, the sections were then blocked with 5% BSA and incubated overnight at 4°C with primary antibodies (Fn: 1:200, p16^INK4a^: 1:50), followed by incubation with secondary antibodies for 1 h at room temperature. After incubation, the sections were washed with TBS, and the specified proteins were visualized using DAB (ZLI‐9018, ZSGB‐BIO). Nuclei were counterstained with hematoxylin, and upon dehydration with xylene, the sections were mounted with neutral resin. The expression levels of FN and p16^INK4a^ were quantified utilizing ImageJ.

### 4.7 Immunofluorescence Staining

For γH2AX staining, the paraffin‐embedded renal tissue sections were initially blocked with 5% BSA containing 0.3% Triton X‐100. Subsequently, they were incubated overnight at 4°C with the primary antibody (γH2AX: 1:100), followed by incubation with the secondary antibody for 1 h and counterstaining with DAPI for nuclear visualization. Regarding p‐AMPKα staining, after the primary antibody (1:100) and secondary antibody incubation, the sections underwent an additional incubation step with *Lotus tetragonolobus* lectin (LTL) diluted at 1:100 for 2 h before DAPI nuclear staining. As for CAV1 (1:200), LC3B (1:100), SQSTM1/p62 (1:50), and Megalin (1:1000), a double‐label multiplex immunofluorescence kit was utilized according to the manufacturer's instructions (Abiowell Biotechnology, AWI0692).

### 4.8 Transmission Electron Microscopy

The renal tissue was immersed in a 2.5% glutaraldehyde solution (Servicebio, G1124) for fixation and then transferred to the transmission electron microscopy laboratory in the Pathology Department of the Second Xiangya Hospital, Central South University, for further specimen processing. The specimens underwent examination and photography using a JEOL JEM‐1400 system.

### 4.9 Senescence‐Associated β‐galactosidase (SA‐β‐gal) Staining

Frozen sections of renal tissue or cells were fixed at room temperature for 15 min and subsequently stained using the SA‐β‐gal kit (C0602, Beyotime, China) and then incubated at 37°C overnight. Finally, the specimens were examined and imaged under a light microscope.

### 4.10 Measurement of Serum Creatinine and Urea Nitrogen

Serum creatinine (C011‐2‐1, Nanjing Jiancheng Bioengineering Institute) and blood urea nitrogen (C013‐2‐1, Nanjing Jiancheng Bioengineering Institute) levels were measured using dedicated assay kits, adhering strictly to the manufacturer's protocols for experimental procedures and calculations.

### 4.11 Co‐immunoprecipitation

HK‐2 cells were co‐transfected with Flag‐tagged CAV1 and HA‐tagged CaMKK2 for 48 h. The cells were then lysed using immunoprecipitation lysis buffer (catalog 87787; Thermo Fisher Scientific) containing protease and phosphatase inhibitors. The lysates were incubated overnight at 4°C with Anti‐Flag Affinity Gel (20585ES; Yeasen Biotechnology) or HA antibodies. The precipitated complexes were subsequently subjected to Western blot analysis using anti‐Flag and anti‐HA antibodies.

### 4.12 GST Pull‐down

The BL21 strain was induced to express and purify the SUMO‐CAV1‐His and GST‐CAMKK2 fusion proteins. Sepharose 4B beads conjugated with GST‐CAMKK2 were suspended in an appropriate volume of buffer, and 20 μL of the SUMO‐CAV1‐His protein solution was added. GST‐coupled Sepharose beads were used as a negative control. The mixture was incubated on a shaker for 4–8 h at 4°C. Interaction between the proteins was then detected by Western blot analysis.

### 4.13 Single‐Cell Transcriptomic Data

Single‐cell RNA sequencing (scRNA‐seq) data of fetal, adult, and aging mice were obtained from the GEO database (http://www.ncbi.nlm.nih.gov/geo, GSE 198832). The RNA‐Seq analysis procedure (including cell annotations) was described by Wang et al. (2023). The DotPlot function was used to visualize the mRNA expression of *Cav1* in different groups.

### 4.14 Statistical Analysis

Data analysis was conducted utilizing SPSS version 25.0, and graphical representations were generated using GraphPad Prism, version 8.0, software. The experimental data are depicted as mean ± standard deviation and were analyzed using Student's t test or one‐way ANOVA. All reported p‐values are two‐tailed, with significance denoted by *p* < 0.05.

## Author Contributions

Liya Sun conducted the study, performed data analysis, and drafted the initial manuscript; Lujun Xu, Tongyue Duan, Yiyun Xi, and Zebin Deng contributed to the experimental design and participated in discussions; Shilu Luo and Chongbin Liu edited the final manuscript; Chen Yang and Huafeng Liu provided technique support and research resources; Lin Sun was responsible for the overall study design, coordination, and manuscript revision. All authors have reviewed and approved the final manuscript.

## Conflicts of Interest

The authors declare no conflicts of interest.

## Supporting information


Appendix S1.

## Data Availability

Data supporting the findings of this study are available from the corresponding author upon reasonable request.
