# Supplementary material for: CAV1 Exacerbates Renal Tubular Epithelial Cell Senescence by Suppressing CaMKK2/AMPK‐Mediated Autophagy
Source: Aging Cell. 2025 Jan 30;24(5):e14501. doi: 10.1111/acel.14501 (PMC12073896; doi:10.1111/acel.14501)
Supplement: Supplementary file 1 — Appendix S1. [file ACEL-24-e14501-s001.docx]

**CAV1 exacerbates renal tubular epithelial cell senescence by suppressing CaMKK2/AMPK-mediated autophagy**

Liya Sun^1^, Lujun Xu^1^, Tongyue Duan^1^, Yiyun Xi^1^, Zebin Deng^2^, Shilu Luo^1^, Chongbin Liu^1^, Chen Yang^3^, Huafeng Liu^3^, Lin Sun^1^

^1^Department of Nephrology, The Second Xiangya Hospital of Central South University, Key Laboratory of Kidney Disease and Blood Purification, Changsha, Hunan 410011, China.

^2^ Department of Urology, The Second Xiangya Hospital at Central South University, Changsha, Hunan 410011, China.

^3^Guangdong Provincial Key Laboratory of Autophagy and Major Chronic Non-Communicable Diseases, Key Laboratory of Prevention and Management of Chronic Kidney Disease of Zhanjiang, Institute of Nephrology, Affiliated Hospital of Guangdong Medical University, Zhanjiang, China.

Correspondence to: Lin Sun, Department of Nephrology, The Second Xiangya Hospital of Central South University, Key Laboratory of Kidney Disease and Blood Purification, No.139 Renmin Middle Road, Changsha, Hunan 410011, China

E-Mail: sunlin@csu.edu.cn

**Figure S1**

**
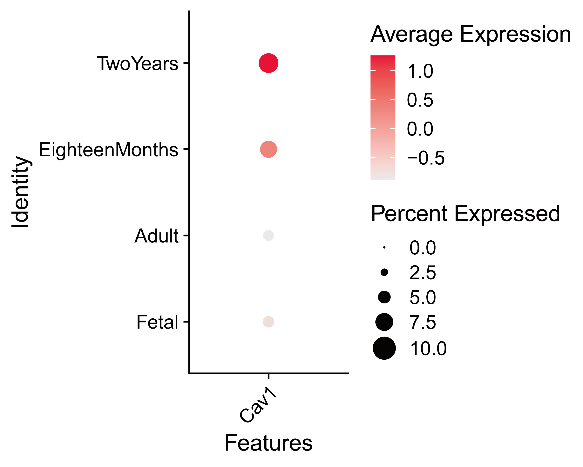
**

Single-cell RNA sequencing data were obtained from the GEO database (http://www.ncbi.nlm.nih.gov/geo, GSE 198832), spanning 4-time points from fetal, adult (8 weeks), 18 months, and 2 years to investigate changes in the expression of Cav1 in proximal tubular epithelial cells of the mouse kidney during aging.

**Figure S2**

**
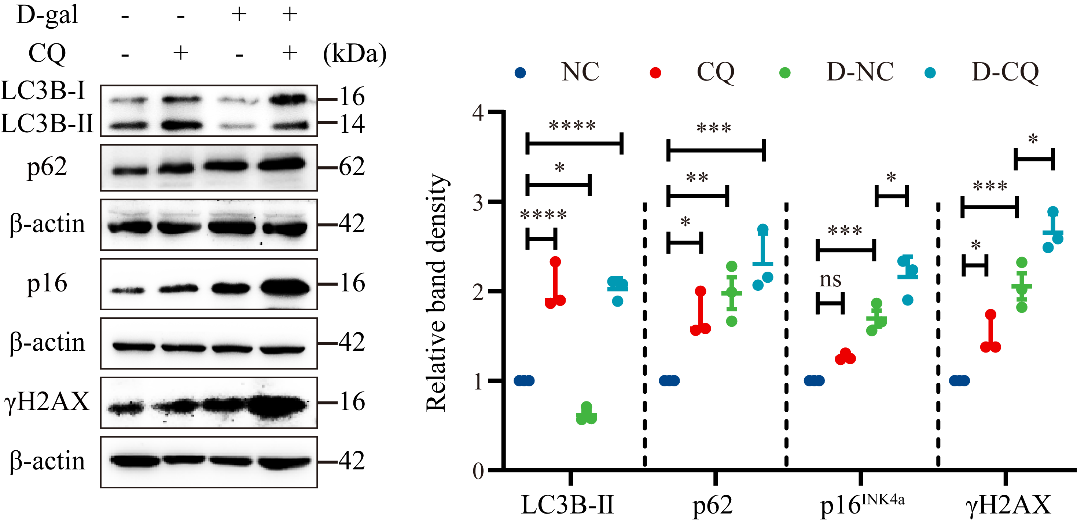
**

Inhibition of autophagy by chloroquine (CQ) exacerbates the senescence process of HK-2 cells.

**Figure S3**

**
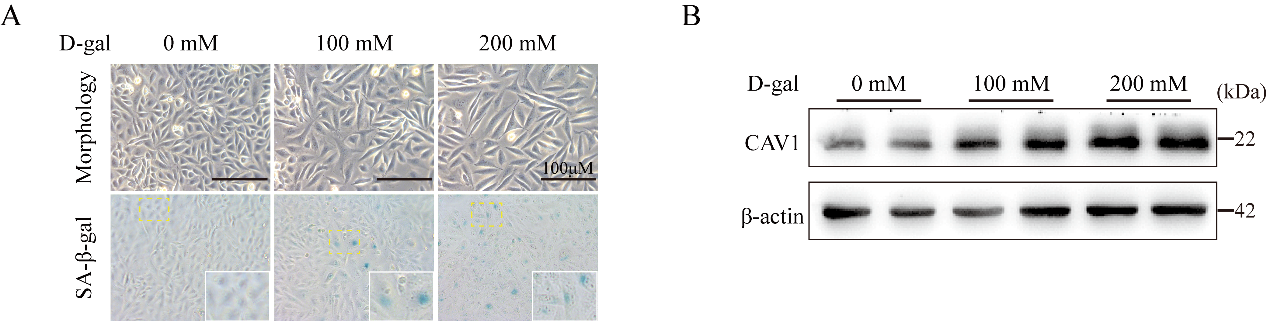
**

(A) Light microscopical analysis of morphological changes and SA-β-gal staining of HK-2 cells after D-gal treatment. (B) Western blot analysis of CAV1 protein expression in HK-2 cells with D-gal treatment at different concentrations.

**Figure S4**


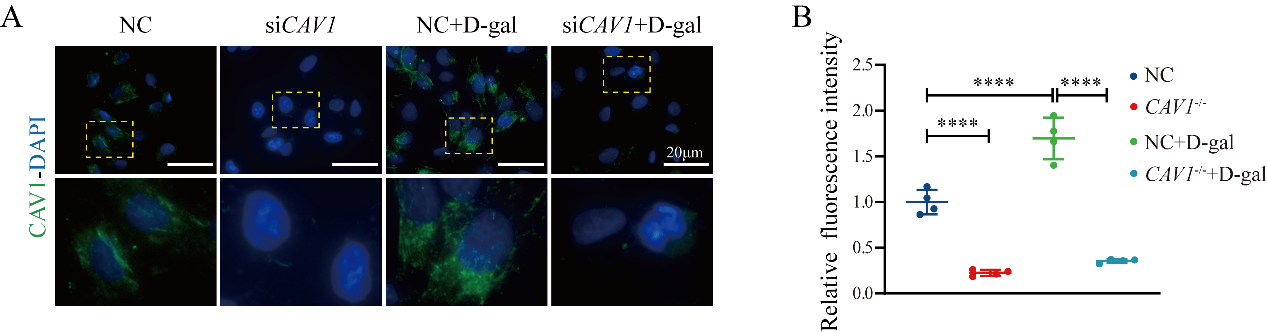


(A) IF staining analysis of CAV1 expression and localization in HK-2 cells. (B) Semiquantitative analysis of relative mean fluorescence intensity of CAV1 (n=4).

**Figure S5**


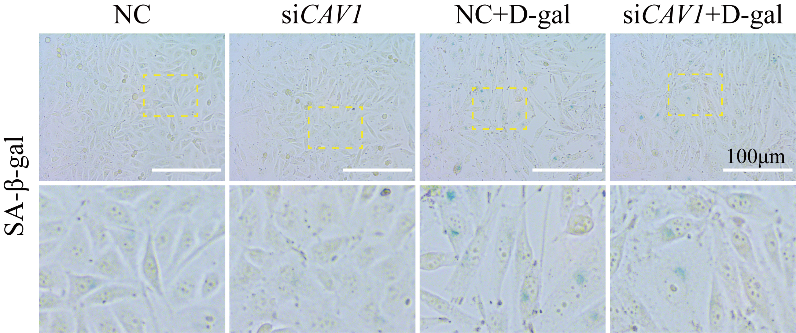


SA-β-gal staining of D-gal-treated HK-2 cells.

**Figure S6**


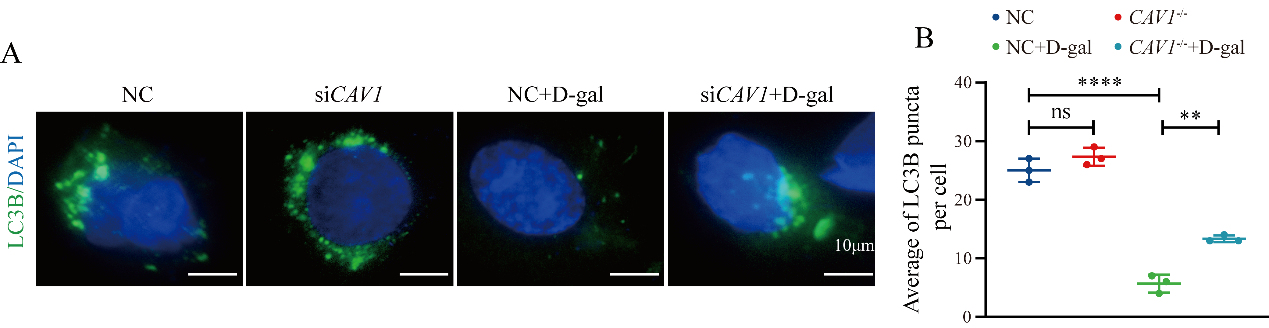


(A) IF staining analysis LC3B expression: LC3B (green), nuclei (blue). (B) Quantitative analysis of LC3B fluorescent dots.

**Figure S7**


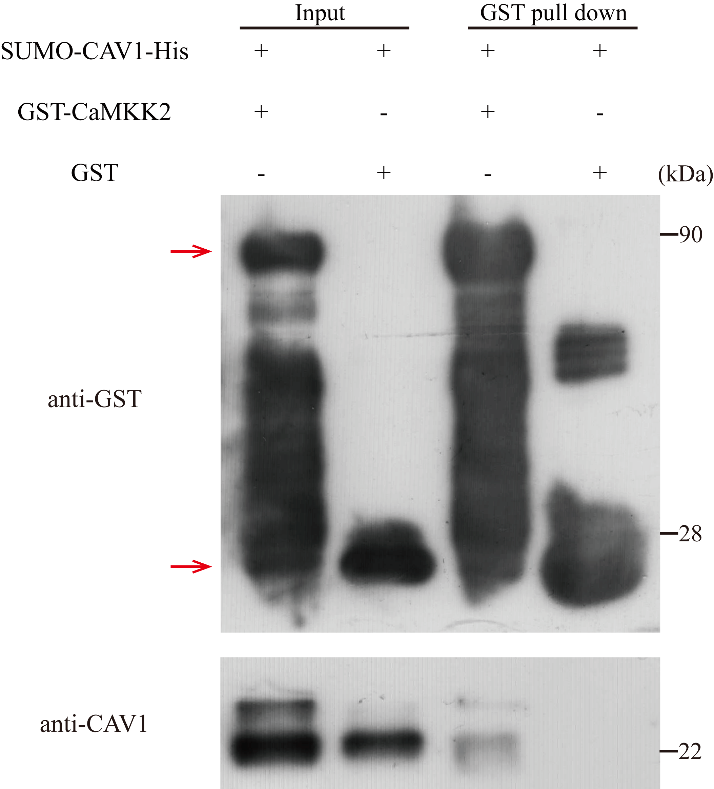


GST pull-down assay demonstrates that CAV1 directly binds to CaMKK2.
